# Bactericidal activity of green tea extracts: the importance of catechin containing nano particles

**DOI:** 10.1038/srep19710

**Published:** 2016-01-28

**Authors:** Judy Gopal, Manikandan Muthu, Diby Paul, Doo-Hwan Kim, Sechul Chun

**Affiliations:** 1 Department of Bioresource and Food Science, Konkuk University, Seoul 143-701, Korea; 2 Environmental Microbiology, Department of Environmental Engineering, Konkuk University, Seoul 143-701, Korea

## Abstract

When we drink green tea infusion, we believe we are drinking the extract of the green tea leaves. While practically each tea bag infused in 300 mL water contains about 50 mg of suspended green tea leaf particles. What is the role of these particles in the green tea effect is the objective of this study. These particles (three different size ranges) were isolated via varying speed centrifugation and their respective inputs evaluated. Live oral bacterial samples from human volunteers have been screened against green tea extracts and macro, micro and nano sized green tea particles. The results showed that the presence/absence of the macro and mico sized tea particles in the green tea extract did not contribute much. However, the nano sized particles were characterized to be nature’s nano stores of the bioactive catechins. Eradication of these nano tea particles resulted in decrease in the bactericidal property of the green tea extracts. This is a curtain raiser investigation, busting the nano as well as green tea leaf particle contribution in green tea extracts.

Tea grows primarily in tropical and temperate regions of Asia which mainly include China, India, Sri Lanka and Japan as well as several African and South-American countries. Green, white, black and oolong teas all originate from the same source, that is, the leaves of *C. sinensis* plant. It’s the degree of fermentation that results in the classification of tea. While green tea is unfermented; white tea, yellow tea and oolong tea are semi-fermented. Black tea is fully fermented^1^,^2^ and dark tea (limited to areas of China and Japan is post-fermented^3^,^4^,^5^. The chemical composition of tea leaves includes: polyphenols^6^, caffeine (approximately 3.5% of the total dry weight), theobromine (0.15–0.2%), theophylline (0.02–0.04%) and other methylxanthines, lignin (6.5%), organic acids (1.5%), chlorophyll (0.5%), theanine (4%) and free amino acids (1–5.5%), and numerous flavor rich compounds^7^. Besides these, flavones, phenolic acids and depsides, carbohydrates, alkaloids, minerals, vitamins and enzymes^8^ are also present. Tea also contains flavanols, mainly quercetin, kaempferol, myricetin, and their glycosides. Due to the processing technique which involves no fermentation, in green tea all these components are well preserved, especially the polyphenols. The most favorable effects of green tea are accredited to the polyphenols in green tea. These predominantly are the catechins, that are equivalent to 25–35% of the dry weight of green tea leaves^9^,^10^,^11^. Green tea extracts are rich in flavanols and their gallic acid derivatives, namely, (+)-catechin, (−)-epicatechin (EC), (+)-gallocatechin (GC), (−)-epicatechin gallate (ECG), (−)-epigallocatechin (EGC), and (−)-epigallocatechin gallate (EGCG)^12^. In addition, they contain a range of natural flavor-rich components such as terpenes, oxygenated terpenes, sesquiterpenes and organic acids

Of the catechins, epigallocatechin-3-gallate and epicatechin-3-gallate are the most effective antioxidant compounds. Other active components in the extract include the other catechins such as epicatechin and epigallocatechin. Among these, epigallocatechin-3-gallate is the most bioactive and the most studied. EGCG, which is the most abundant catechin in green tea, accounts for 65% of the total catechin content. A cup of green tea may contain 100–200 mg of EGCG^13^. Catechin and gallocatechin are present in trace amounts^14^.

Green tea and its constituent catechins are established for their antioxidant properties, encompassing applications in various diseases associated with reactive oxygen species (ROS). This includes cancer, cardiovascular and neurodegenerative diseases. The catechins have been shown to be more effective antioxidants than Vitamins C and E^15^, and their order of effectiveness as radical scavengers is in the order of ECG > EGCG > EGC > EC > catechin. The metal-chelating properties of green tea catechins are also important contributors to their antioxidative activity^16^,^17^,^18^.

Several epidemiological studies as well as studies in animal models have confirmed the defense that green tea can offer, against various cancers such as those of the skin, breast, prostate and lung^19^,^20^. In addition to its cancer chemopreventive properties, green tea and EGCG have been shown to be anti-angiogenic (prevention of tumor blood vessel growth)^18^ and anti-mutagenic^21^,^22^. Green tea has also proved to be hypocholesterolemic^23^ and anti atherosclerotic plaque forming^24^. Among age-associated pathologies and neurodegenerative diseases, green tea has been shown to offer significant protection against Parkinson’s disease, Alzheimer’s disease and ischemic damage^25^. Green tea has also displayed anti-diabetic effects in animal models^26^ and promotes energy expenditure. Other health beneficial attributes include anti-bacterial^27^, anti-HIV^28^, anti-aging^29^ and anti-inflammatory activity^30^. Due to high content of antioxidants, green tea functions as dietary supplement and ingredient in care cosmetics, shampoos, sweet waters, masks and anti-aging emulsions^31^,^32^.

All the above work and reports so far focus only on the green tea extracts. Green tea preparation involves adding boiled water to green tea leaves or a green tea bag and allowing the seeping of the active components from the leaves into the water. The tea bags are then after 3–4 min removed from the cup, or when using whole green leaves, the leaves are filtered using a sieve and the extract is consumed. This extract is the one that has been researched and whose potential widely published. The current work looks deep into this extract, which was observed to contain suspended particles from the green tea leaves. Are these suspended particles playing a positive or deterrent role in the well established bioactivity of green tea ? This work resolves this question by assessing one of the well established properties of green tea, which is antimicrobial activity, against oral microflora. The reason for choosing oral microflora is that this is the first place of contact of the green tea extract when consumed. The individual role of the extract alone has been studied. In addition to this, the effect of the macro particles, the microparticles and nano green tea particles were studied. Moreover, the extracts rid of the particles were also investigated for the first time.

## Materials and Methods

### Chemicals

Three kinds of pure green tea (tea bags), the first two were popular Korean based brands and the third one, a well known international brand. First green tea was iced green tea (emart.kr), the second is Boseong green tea and the third one is Lipton green tea. These were purchased from a local supermarket in Seoul, Korea. Millipore water was used for all experiments. Standards of total phenolic acids (gallic acid) were procured from MP Biomedicals, LLC, Illkirch, France and of flavanoids (rutin hydrate) from abcam Biochemicals, Cambridge Science Park Cambridge CB4 0FL UK. Trolox was purchased from Enzo Life sciences Inc., Farmingdale, NY. 2,2-dyphenyl-1-picrylhydrazyl (DPPH) was obtained from Sigma Aldrich Company, St Louis, MO 63103, USA. The Folin- Ciocalteu’s phenol reagent and aluminium chloride (AlCl3) were from MP Biomedicals, LLC, BP 50067 Illkirch, France. All other solvents and chemicals were of analytical grade.

### Preparation of Green tea samples

#### Green tea extract

The three green teas, henceforth will be represented as GT 1, GT 2 and GT 3. GT 1 was iced green tea and so was extracted in cold water (0 °C) while GT 2 and GT 3, were extracted via the general hot water extraction procedure. 100 mL of sterile water was brought to rolling boil or to 0 °C and the respective tea bags were immersed in it and keep for seeping for 4 min. After 4 min the tea bags were removed and the extract was stored at 4 °C for further use.

#### Green tea particles

The green tea particles suspended in the green tea extract was separated via differential centrifugation (Beckman Coulter Avanti J-25, USA) at 5000 rpm, 10,000 rpm and 20,000 rpm for 5 min, 10 min and 2 h respectively. The idea behind this is to separate the macro sized tea particles from micro sized and lastly the nano sized ones. For GT 1 at 5000 rpm, the 5K-P particles and the supernatant with the particles removed (5K-S) were coded. The particles were suspended in 10 mL sterile water. Similarly 10K-S and 10K-P were obtained from 10,000 rpm and 20K-S and 20K-P resulted following 20,000 rpm centrifugation. For GT-2 and GT-3 similar test variables were also generated. These codes will be used in the results section.

#### Characterization of the green tea samples

Test variables (extract and particles) were characterized using a Nanodrop ND-1000 v 3.3.1 spectrophotometer, (Nanodrop Technologies, Inc., Wilmington, USA). The absorbance was scanned from 220-700 nm. The 5K-P, 10K-P and 20K-P particles were characterized employing FTIR (Shimadzu FTIR-8300 spectrometer, San Diego, CA, USA) using KBr pellets. For FTIR the samples were dried in an oven and the precipitated particles were used for analysis. The size and morphology of the particles was determined using Field Emission Scanning electron microscope (FE-SEM).

The total phenolic content in the extracts and in the green tea particles isolated from the extracts of the three green teas was determined by the spectrophotometric method^33^. The reaction mixture consisted of 0.5 mL of the extract/particles, 2.5 mL of 10% Folin-Ciocalteu’s reagent dissolved in water and 2.5 mL 7.5% NaHCO_3_. Blank was concomitantly prepared, by replacing the extract with water. The samples were incubated in a waterbath at 45 °C for 45 min and the absorbance measured using spectrophotometer at λmax = 765 nm. All samples were prepared in triplicate for each analysis and the mean value of absorbance was obtained. The same procedure was repeated for the standard solution of gallic acid and the calibration line was construed. Based on the measured absorbance, the concentration of phenolics was read (mg/mL) from the calibration line and the content of phenolics in the green tea extracts and green tea particles was expressed in terms of gallic acid equivalents (mg of GA).

The content of flavonoids was determined using the spectrophotometric method^34^. The sample contained 1 mL of the green tea extract/particles and 1 mL of 2% AlCl_3_ solution dissolved in ethanol. The samples were incubated for an hour at room temperature and read at λmax = 415 nm. The same procedure was repeated for the standard solution of rutin and the calibration line was construed and the flavonoid content in the extracts/particles expressed in terms of rutin equivalent (mg of RU).

The DPPH assay was conducted according to Brand-Williams *et al.* with some modifications. 190 μL of 0.12 mM DPPH solution was mixed with 10 μL of each test sample solution in 96-well plates. After 30 min, the decrease of absorbance was measured at 517 nm using a microplate reader. The standard curve was prepared with 1 μM Trolox. Results were expressed in μM of Trolox. Antioxidant activity % was calculated using the formulae^35^,^36^:

1− [Absorbance sample/Absorbance control] x 100

### Antimicrobial activity

#### Streptococcus mutans

One of the promising bioactivities of green tea, namely, antimicrobial activity was tested against a standard oral pathogen, *S.mutans*. *Streptococcus mutans* 11823 (ATCC 25175) was purchased from Korean culture centre of microorganisms, Seoul, South Korea. All the initial optimization studies and the standardization protocols were carried out using this dental pathogen as the model organism, grown in Brain heart infusion (BHI) broth. 300  μL/L concentration of GT 1-0, GT 2 and GT 3–0 and their respective GT1 5K-S, 5K-P, 10 K S, 10K-P, 20K-S and 20K-P were incubated with *S. mutans* for 12 h and the bacterial growth was measured at 600 nm using *ND*-1000 v 3.3.1 spectrophotometer, (Nanodrop Technologies, Inc., Wilmington, USA). Control (no addition of green tea extract) was also maintained. The total viable count, indicating the number of bacteria that survived after interaction with the different green tea variables, was enumerated by plate count method. The TVC was represented as cfu/mL (colony forming unit /mL)^37^.

0 μL, 50 μL, 100 μL, 200 μL, 300 μL and 500 μL concentrations of GT-0 (extract with no particles removed) and GT-5K-S of GT 1, GT 2 and GT 3 were also incubated with *S.mutans* to obtain the minimum inhibitory concentration.

#### Dental real sample testing

**Figure 1:**
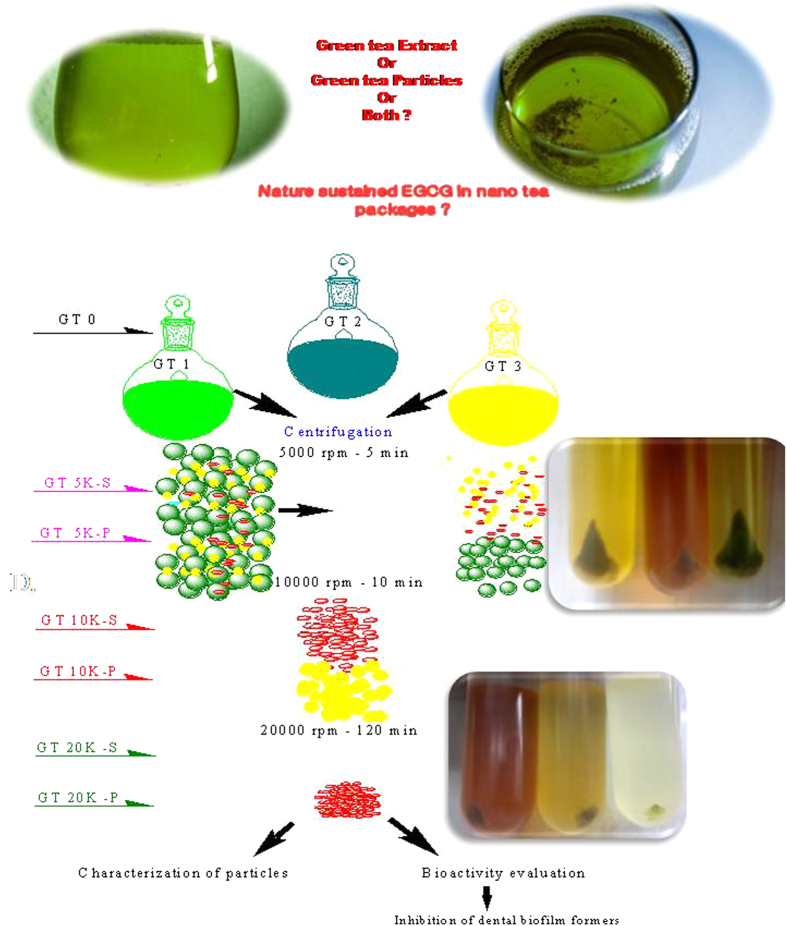
Schematic showing work flow followed in the study.

Five healthy volunteers were chosen at random, they were asked to collect overnight grown biofilm from their teeth in the early morning using sterile brushes. Samples were coded Volunteer (V) V1, V 2, V3, V4 and V5. The biofilm from the brush was dislodged into 15 mL sterile 1% BHI medium. Methods were carried out in accordance with the approved guidelines. Experimental protocol was approved by the ethical committee of the University Hospital. The informed consent was obtained from all the five subjects for the collection of the oral biofilm. To 5 mL of each dental biofilm sample, 200 μL of the respective green tea test variable was added and inoculated for 3 h at 32 °C in the shaking incubator. GT-0, GT 5K-S and GT-20K-S were used for GT 1, GT 2 and GT 3. Control sets were also maintained for each volunteer. After 3 h the samples were plated in BHI agar and the total viable counts of the dental bacteria surviving the green tea attack were enumerated by plate count method. The dental bacteria after the green tea attack were viewed and imaged using a fluorescence microscope (Axiovert 2000,Carl Zeiss, Germany). To 500 μL of the interacted sample, 100 μL of acridine orange (0.1% solution in distilled water) was added and incubated in dark for 10 min. After 10 min, the unbound stain was removed by centrifugation at 5000 rpm for 10 min. This washing was repeated thrice and the acridine orange stained cells were finally suspended in 500 μL sterile distilled water. Then 10 μL of the respective cell suspensions were laid on glass slides and covered with a cover slip and viewed using a fluorescence microscope. Acridine orange, a fluorescent dye, differentially stains single stranded RNA and double stranded DNA, fluorescing orange when intercalated with the former and green while complexing with the latter when observed under a Axiovert 2000 (Carl Zeiss, Germany) inverted epifluorescence microscope (excitation filter BP 490; barrier filter O 515). Thus, the number of orange fluorescing cells depicts the actively metabolizing cells and the green fluorescing cells the dead cells^38^. The dental bacteria prior to and after green tea interaction were imaged using a Olympus FluoView™ FV1000 confocal laser scanning microscope (CLSM), OLYMPUS AMERICA INC. Corporate Center Drive, Melville, NY, USA and FE-SEM. Figure 1 displays the schematic flow of the experimental methodology used in the study.

## Results

### Bioactivity Testing- Anti-Streptococcal activity

**Figure 2:**
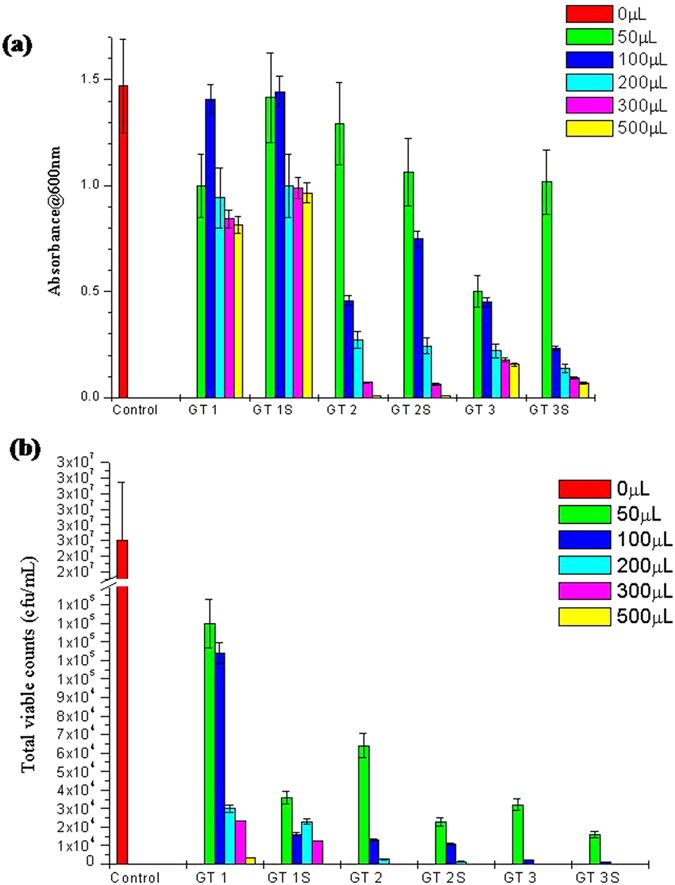
Optimization of the concentration effect of the test variables for antimicrobial activity using (**a**) Spectrphotometric optical density and (**b**) Total viable count methods.

The three different green tea’s and their extracts and particles obtained at 5000 rpm and 10000 rpm and 20000 rpm were studied for their antibacterial properties against the dental pathogen, *S.mutans*. In order to optimize the concentration of the green tea extract for further experiments, six different concentrations (0 μL, 50 μL, 100 μL, 200 μL, 300 μL and 500 μL) of GT-0 (without removing particle) and 5K-S from GT 1, GT 2 and GT 3 were tested against *S. mutans.* Figure 2 shows the results of this study. As observed from Fig. 2(a), the spectrophotometric method revealed that in case of GT-0 and 5K-S with respect to all three green teas, the anti-streptococcal activity increased with increasing concentration. 300 μL concentrations were observed to show optimal antimicrobial activity and hence distinguished as the optimized concentration for all other experiments in this study. Thus, from now on, all experiments were performed using 300 μL concentrations of the green tea variables. Figure 2(b) displays the results obtained through antimicrobial assessment using total viable count method. The plate count results correlated with that observed in case of the spectrophotometer based turbidity measurements. Another interesting observation observed from these results is the marginal improvement in antimicrobial activity observed on removal of the particles at 5000 rpm (5K-S) compared to GT-0. Compared to GT 1-0, GT 1-5K-S showed increased antibacterial activity, similarly in case of GT 2 and GT 3. Moreover, GT 3 was observed to exhibit the highest antibacterial activity and GT 1 the least antibacterial activity.

**Figure 3:**
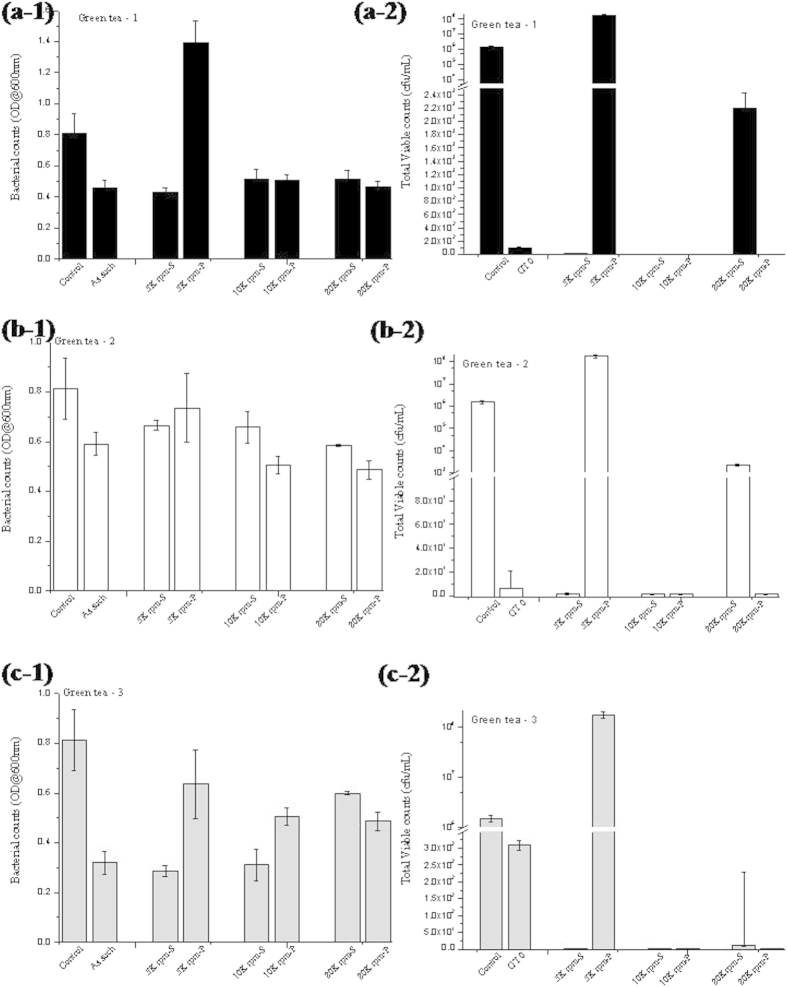
Graph comparing the antibacterial activities of the various test components of (**a**) GT 1, (**b**) GT 2 and (**c**) GT 3 via (**a,b,c -1**) Spectrophotmetric method and (**a,b,c -2**) Total Viable count method.

Since the removal of the particles from the green tea showed changes in the bioactivity of the green tea extract, it was proposed to do a detailed investigation on studying this effect. A systematic three step centrifugation process, at 5000 rpm, 10,000 rpm and 20,000 rpm meant to separate the largest, smaller and still smaller particles from the extract was carried out. The extract rid of the large particles (5K-S), small particles (10K-S) and fine particles (20K-S) and the particles (5K-P, 10K-P and 20K-P) were studied for their interaction with *S. mutans* and their individual antibacterial activities. Figure 3 gives the results of this study. As observed in Fig. 3(a-1,a-2), both the turbidity based method and the plate count method, confirmed, that with respect to GT 1, the removal of the 5K-P resulted in enhanced (marginal enhancement) antibacterial activity of the extract (5K-S) compared to GT 1-0. The removal of 10K-P did not result in any further enhancement and 10K-S was usually found to be similar to the 5K-S activity. However, the removal of 20K-P’s resulted in a distinct loss of antimicrobial activity in 20K-S. This observation was observed to be evident in case of GT 2 (Fig. 3(b-1,b-2)) and GT 3 (Fig. 3(c-1,c-2)). Although the trend had varying degrees of variation from GT 1 to GT 2 to GT 3, but the fact that the removal of the large green tea particles from the extract marginally increased the antibacterial property of the green tea extract and that the eradication of the smaller green tea particle fractions from the extract lead to a distinct decrease in the antibacterial activity of the green tea remains unquestioned.

However, it is important to emphasize here that neither the 5K-P, 10K-P nor 20K-P particles showed enhanced stand alone antibacterial activity higher than the extracts. 5K-P particles showed nil antibacterial activity, 10K-P showed a limited extent of antibacterial activity, while the 20K-Ps showed some activity amidst the other two counterparts.

### Real time testing of dental bactheria

**Figure 4:**
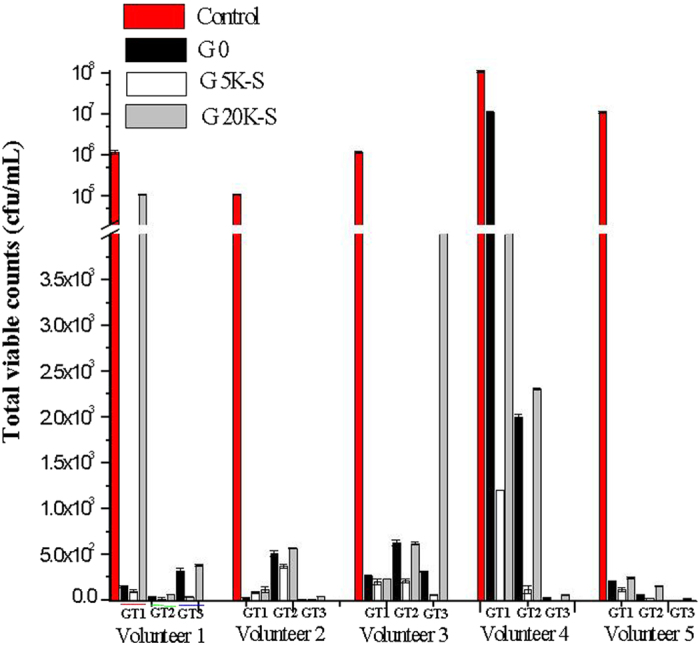
Results showing the successful dental anti-bacterial effect demonstrated on the real samples obtained from five human volunteers.

In order to evaluate this variation in the bioactivity of the green tea extracts rid of the green tea particles in real time systems, the extracts were put up against real samples of dental biofilm from five different human volunteers. GT 0, 5K-S and 20K-S of GT 1, GT 2 and GT 3 were tested. Figure 4 gives the total viable counts following the plate count method, indicating the bacteria that survived the green tea interaction. It was interesting to observe that despite the complexity of the sample, compared to GT 0, 5K-S showed enhanced antibacterial activity, while 20K-S showed decreased antibacterial activity with respect to all three green teas.

**Figure 5:**
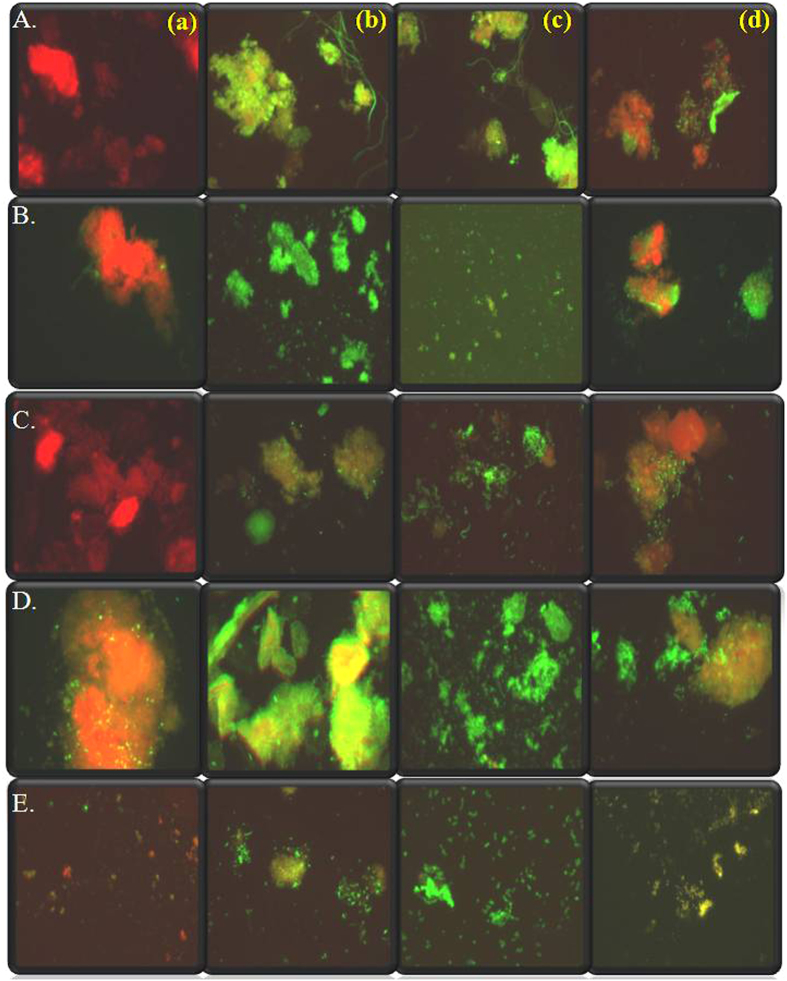
Epifluorescence micrograph imaging the live/dead nature of the oral biofilm from (**A**) Volunteer 1 (**B**) Volunteer 2 (**C**) Volunteer 3 (**D**) Volunteer 4 in (**a**) Control (no GT component) (**b**) GT 0 (**c**) 5K-S (**d**) 20K-S belonging to GT 3.

Fluorescence imaging of the cells using acridine orange, aids in the visualization and differentiation between live/dead cells after treatment. We imaged the dental biofilm samples from the five volunteers prior to and subsequent to interaction with GT 0, 5K-S and 20K-S and control. Figure 5 displays the fluorescence results showing effect of the green tea variables on volunteer 1 (A), volunteer 2 (B), volunteer 3 (C), volunteer 4 (D) and volunteer 5 (E). As observed from Fig 5(a), the control images in A, B,C,D and E show predominantly orange fluorescence indicating presence of actively metabolizing live dental bacteria. As observed from the image, the dislodged biofilm from the teeth retained their biofilm identity and appear as microbial mats in case of the control. The GT 0 treatment resulted in the well expected killing effect expected of green tea (Fig. 5(b)) in case of all the volunteers. The green fluorescing areas (dead cells) predominated, with areas of orange fluorescence coexisting amidst. Panel (c) depicts the results from the 5K-S supernatant of GT 3, where the largest green tea particles were removed from the extract. As observed from the fluorescence microscopic images, there was no orange fluorescence observed and complete green fluorescence indicating total annihilation of the dental bacteria/biofilm. Also it was interesting to observe that no longer biofilm patches or mats were observed, the biofilm mats were disintegrated and all that is seen are scattered cell clumps in all test samples (A(c), B(c).C(c).D(c) and E(c). Finally, Fig. 5(d) gives the 20K-S interaction results, a significant drop in the killing effect of the green tea extract is evident. The occurrence of the orange fluorescing live bacterial cells indicates a decrease in the antimicrobial activity of the extract on removal of the nano green tea components. Although green fluorescing dead cells are also observed, the ratio to the live/dead cells seemed altered. These results confirmed the trend reported via the spectrophotometric method and plate count method of antimicrobial activity of the various test green tea components.

**Figure 6:**
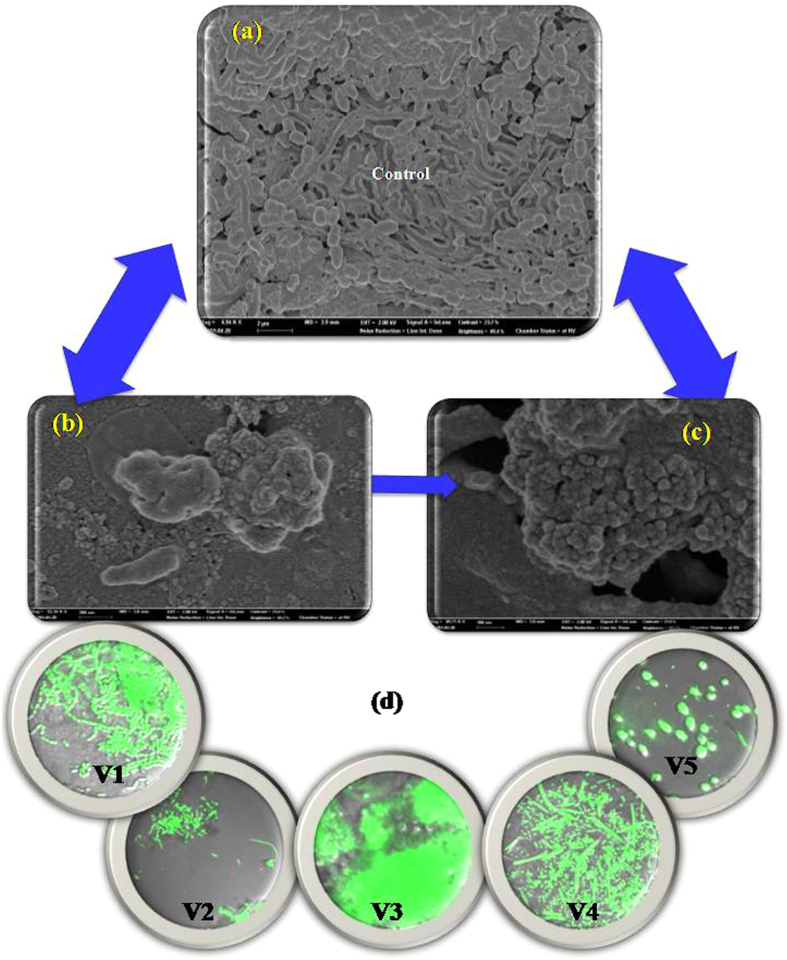
FE-SEM images of dental sample from V4 (**a**) control (untreated) (**b,c**) GT 3 treated, showing extensive damage following incubation with green tea. (**d**) shows the CLSM images of the cells, the fluorescing cells representing the dead cells in the five different volunteers following treatment with GT 3 20K-P’s.

FE-SEM observation of the control and 5K-S interacted dental bacteria is given in Fig. 6 (a–c). As observed from the figure, the control (Fig. 6(a)) shows well developed biofilm (Volunteer 4), while the GT 0 (Fig. 6(b)) of GT 3 and 5K-S (Fig. 6(c)) interaction resulted in damaging the cells and the biofilm, as indicated by the cell debris. Images captured using CLSM (d) show that a vast majority of the dental bacteria from all five volunteers were killed by GT 3 (5K-S). GT 3-GT 0 and 5K-S were found to be the most promising against the dental bacteria from all volunteers.

### Characterization of extract and particles

With the results from the bioactivity testing based on the antibacterial activity of the green tea components showing that the presence and absence of the green tea particles does in fact alter the activity of the extract, it is necessary that the extracts GT 0, 5K-S, 10K-S and 20K-S and then particles 5K-P, 10K-P and 20K-P of the three green teas are characterized elaborately.

### Biochemical characterization

**Figure 7:**
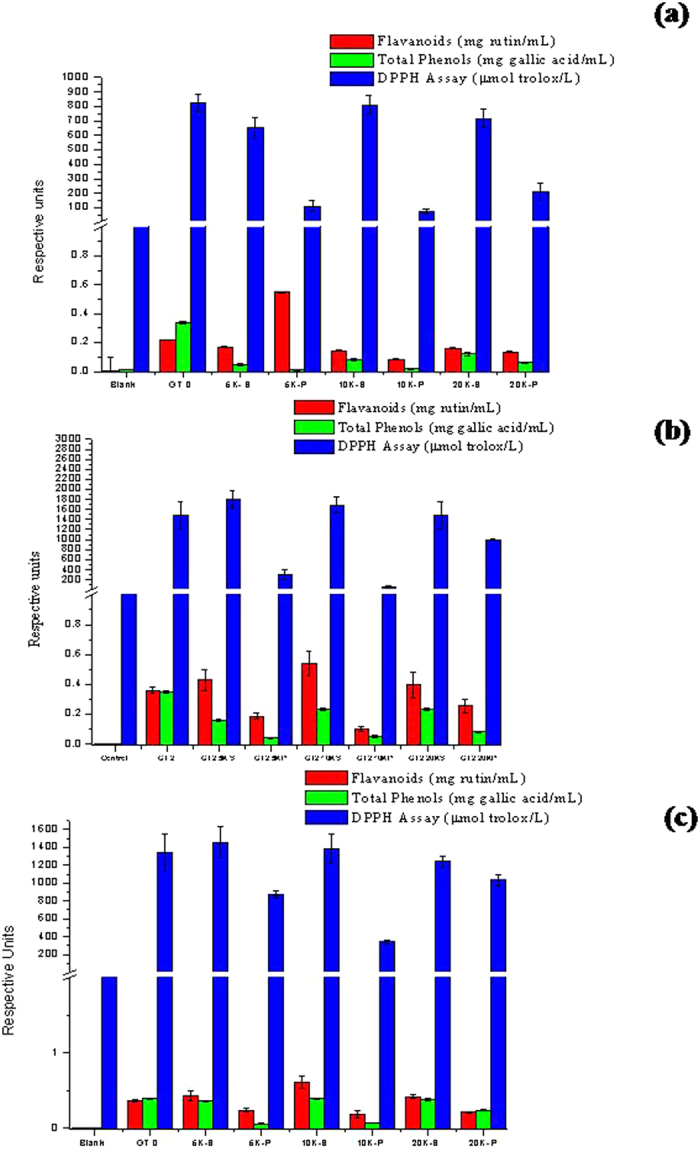
Comparison of the biochemical activities of the (**a**) GT 1 (**b**) GT 2 and (**c**) GT 3 components based on their antioxidant activity (using DPPH), flavonoid content (AlCl3 method) and total phenolics (Folin’s method).

Antibacterial activity is generally governed by total phenolics, flavanoids and antioxidant ability of an extract. All these parameters were studied and compared amidst the extracts and the particles employed in this study. Figure 7(a) shows the results obtained in case of GT 1, in case of flavanoids, as observed from the graph, not much difference was observed in the flavanoid content among GT 0, 5K-S, 10K-S and 20K-S extracts, GT 0 appeared to show marginal increase compared to the rest. However in case of the green tea particles, 5K-P recorded the highest flavanoids content, with 10K-P and 20K-P following in ranges of flavanoids similar to that found in the extracts. In case of GT 2 Fig. 7(b) and GT 3 Fig. 7(c) the flavanoids content was different, 5K-S and 10K-S extracts showed highest flavanoids contents compared to GT 0. The 5K-P and 10K-P particles showed very low flavanoids contents. The exceptions were that the 20K-S extracts showed lesser flavanoids compared to the other extracts and the 20K-P particles showed higher flavanoids compared to the other particles.

With respect to the total phenolics, in GT 1 the highest was found in GT 0, while the rest showed lesser values. However in case of GT 2 and GT 3 it was again observed that a similar trend was observed in case of phenolic content, where all the extracts showed nearly similar phenolic contents. But the 5K-P and 10K-P showed least phenolic contents compared to 20K-P.

In terms of antioxidant activity, GT 1 extracts (GT 0, 5K-S, 10K-S and 20K-S) showed very high antioxidant activity, while 5K-P and 10K-P showed six times lower antioxidant activity. However, it needs to be mentioned here that the 20K-P’s showed significantly higher antioxidant activity compared to the 5K-P and 10K-P. In GT 2 and GT 3 this trend was more pronounced, with the 5K-S and 10K-S exhibiting higher antioxidant activity compared to GT 0. But a distinct decrease in the antioxidant activity was observed in case of 20K-S with a corresponding increase in activity in 20K-P’s of GT 2 and GT 3. It needs to be noted that amidst the three green teas studied, GT 3 recorded higher values of these bioactive compounds closely followed by GT 2 and finally significantly trailing behind was GT 1. This trend strongly correlates with the antimicrobial bioactivity of the green teas which was in the order of GT 3 > GT 2 > GT1.

### FE-SEM analysis

**Figure 8:**
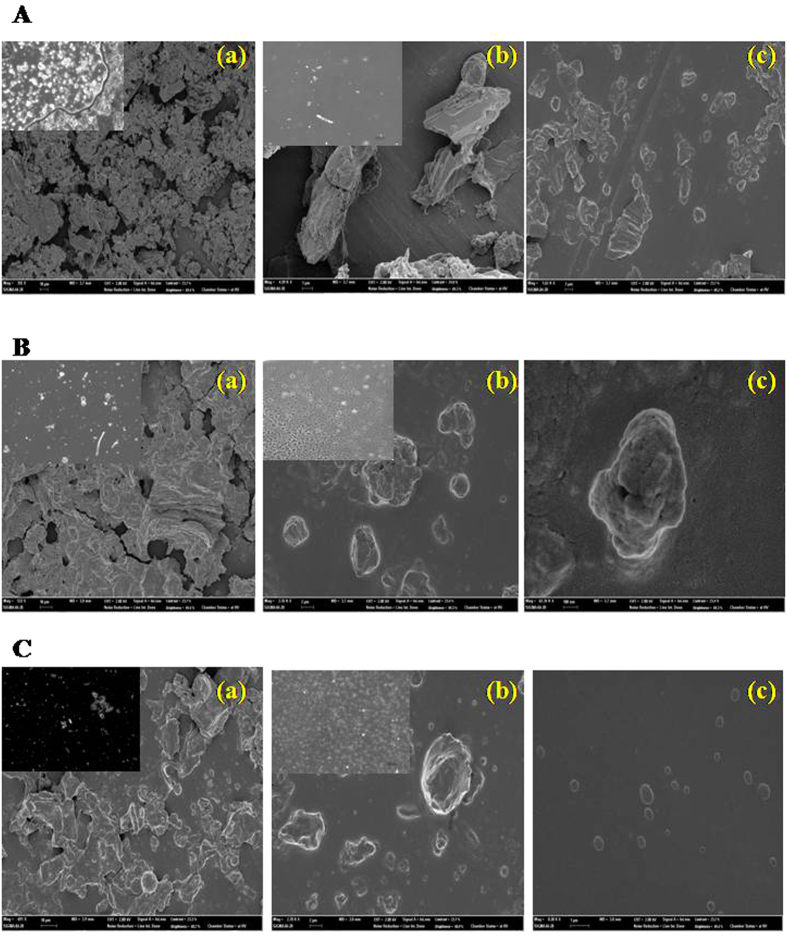
FE-SEM micrographs of (**a**) 5K-P, (**b**) 10K-P and (**c**) 20K-P of GT 1 (**A**), GT 2 (**B**) and GT 3 (**C**), showing morphology and size of the extracted particles. Insets show optical micrographs of the corresponding particles.

The 5K-P, 10K-P and 20K-P particles were imaged using FE-SEM for their morphological details and sizes. Figure 8 gives the 5K-P (a), 10K-P (b) and 20K-P (c) morphologies of the particles from GT 1 (A), GT 2(B) and GT 3 (C). Irregular morphologies with no distinct shape were observed in most cases. As observed from Fig. 8, the 5K-P particles in case of GT 1 and GT 2 were observed to be macrosized, these are the ones we see visibly in our cup of green tea. Table 1 displays their sizes, GT 2 (B) 5K-P particles were the largest (50–80 μm), followed by GT 1(B(a)) which were in the size range of 15–25 μm. GT 3 5K-P particles were relatively smaller in the range of 6–30 μm (Fig. 8(C(a))). As observed in the micrographs the particles were not of fixed sizes, which are expected of such crude unstandardized commercial samples. The 10K-P particles were in the microsized ranges of 4–10 μm in case of GT 1 (A(b)), GT 2 was 2–10 μm (Fig. 8B(b)) and GT 3 in the size range of 0.5–3 μm (C(b)). The 20K-P particles were smaller microsized to nanosized, with GT 1 particles in the 0.5–6 μm regime (A(c)), GT 2 particles (B(c)) were 200 nm to 540 nm and GT 3 (C(c)) particles were the least sized existing in the size regime of 50 nm–300 nm. Thus as shown by these results, 20K-P particles that possessed enhanced bioactive components and showed antioxidant and antibacterial property were observed to exist in the near-nano regime.

### UV-Vis Spectrophotometry

**Figure 9:**
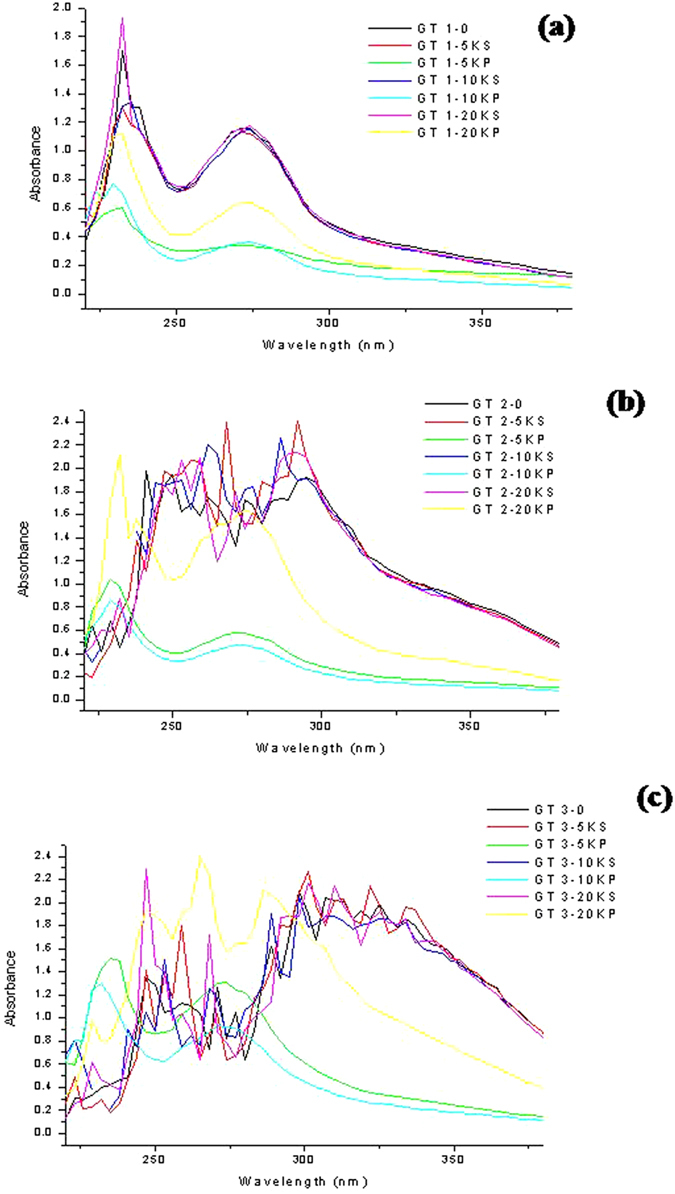
UV- Visible spectra of the various green tea components used as test variables in this study.

Figure 9 gives the UV-Vis spectrum of the extracts and particles characterized for their EGCG contents. Atomssa & Gotlap 2015^39^ have reported the absorbance for the catechin family: EGCG shows an absorbance in the range of 248–361 nm in water with λ_max_ at 273.6 nm; ECG 246- 363 nm λ_max_ at 276.8 nm; the spectral range of EGC in water is 254-378 nm and λ_max_ at 269.6 nm and that of EC is 252-328 nm with λ_max_ at 278.4 nm. As observed in Fig. 9(a) GT-1, we only see the EGCG absorption peak at 273 nm. In terms of EGCG peak no difference was observed within the extracts (GT 0, 5K-S, 10K-S and 20K-S). However, in case of the green tea particles it was observed that the 20K-P particles showed significant increase in the EGCG intensity compared to the 5K-P and 10K-P’s. It was interesting to observe that the 20K-P’s held almost 50% EGCG contained in the extracts.

In case of GT 2, it was observed that the extracts showed the presence of other catechin family peaks in the range of 248-363 nm, as observed from the various peak spikes in this range in Fig. 9(b). The removal of the particles from the extract led to shifts in the peaks. The extracts especially showed distinct shifts. However, in case of the particles 5K-P and 10K-P it was observed that they solely showed EGCG peaks and at low intensity. The emphasis here is the 20K-P particles that showed almost similar intensity catechin peaks compared to the extracts. It was observed that unlike the 5K-P and 10K-P’s the 20K-P’s not only showed EGCG peaks but also the other catechin family peaks, rather similar to the extracts.

GT 3 (Fig. 9(c)), the extracts yielded various peaks, including the catechin peaks. However compared to GT 1 and GT 2, the 5K-P and 10K-P particles themselves showed high intensity EGCG peaks. The trend that the 5K-P and 10K-P’s showed only EGCG peak continued to hold good in GT 3 too. 5K-P showed higher EGCG compared to its GT-1 and GT 2 counterparts, but compared to 10K-P’s of GT 3 it was much lower. The 10K-P of GT 3 showed EGCG contents similar to that in the extracts. The 20K-P’s in this case recorded the highest catechin peaks, excelling the extracts too. Peak shifts were observed similar to those reported within water extraction and solvent extraction in case of the 20K-P’s Vs the extracts. It was interesting to note that the 20K-P peaks were narrower and high intensity. Exceptionally high peak at 269 nm corresponding to EGC was observed in the 20K-P’s. With the general observation that the 20K-P’s contained significantly large amounts of catechins in GT-1, GT 2 and GT 3 were confirmed via these studies.

### FT-IR

**Figure 10:**
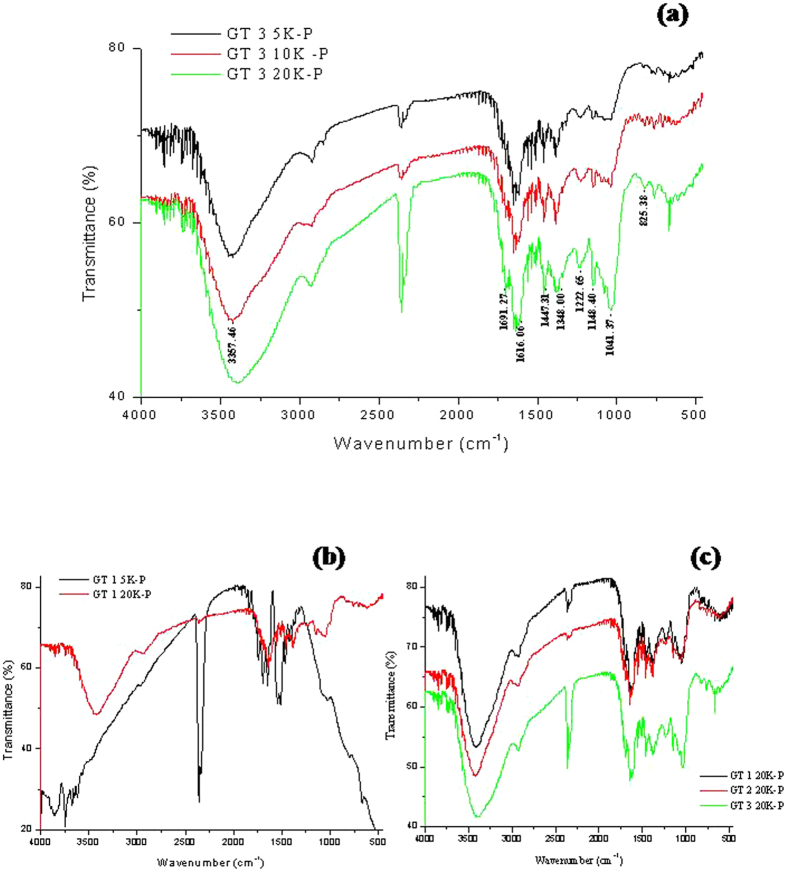
FT-IR results of the 5K-P, 10K-P and 20K-P particles isolated from (**a**) GT 3; (**b**) 5K-P and 20K-P of GT 1 and (**c**) the 20K-P nanoparticles isolated from GT-1, GT 2 and GT 3.

The FT-IR spectrum obtained from the particles 5K-P, 10K-P and 20K-P of GT 3 which showed the maximum antibacterial activity and unique properties is presented in Fig. 10(a). The spectra match the characteristic band of EGCG. Ponnuraj *et al.*, 2015^40^ report EGCG fingerprints at 3357.46 cm^−1^ for O-H group attached to the aromatic ring, 1691.27 cm^−1^ and 1616.06 cm^−1^ strong for C = O group that links the trihydroxybenzoate group and chroman group, 1447.31 cm^−1^ for C-H group present in the Chroman ring, 1348.00 cm^−1^, 1222.65 cm^−1^ for O-C = O group, 1148.40 cm^−1^ for O-H group, 1041.37 cm^−1^ for C-O-C group which links the Chromane ring and trihydroxy benzoate ring and 825.38 cm^−1^ for C-H group in the aromatic ring. It was interesting and supportive to observe that a distinct pattern was observed as a function of increasing centrifugation which corresponds to decreasing particle sizes. At 5K-P the EGCG bands were lowest in intensity, followed by 10K-P and 20K-P showed high intensity bands. This correlates with the results observed in the UV studies too.

Figure 10(b) displays the FT-IR spectra obtained from GT 1 5K-P and 20K-P, In case of GT 1 there wasn’t much of the difference in the EGCG bands between the two particles. These results match that observed in case of the UVspectrophotometric studies too. Figure 10(c) gives the comparative spectra of the 20K-P’s from GT 1, GT 2 and GT 3. The gradient pattern of increased EGCG bands in the order of GT 1 < GT 2 < GT 3 is evident.

## Discussion

The bioactivity of green tea, its benefits is well established and documented. Antibacterial activity of green tea has also been investigated in details. Green tea has been known to prevent dental caries for decades. The active antibacterial component in green tea has been nailed to be EGCG. EGCG has received much attention for its effects on the inhibition of HIV infection and multidrug-resistant *Staphylococcus aureus* infections^27^,^28^,^41^. The most favorable effects of green tea are accredited to the green tea polyphenols, predominantly the catechins, which make up, 25–35% of the dry weight of green tea leaves^9^,^10^,^11^. The tea catechins belong to the family of flavanoids and possess two benzene rings referred to as the A- and B-rings and notorious for their bactericidal effect.

Hubera *et al.*^42^ have evidenced that polyphenolic compounds can interfere with bacterial quorum-sensing. Catechin-copper (II) complexes damage the cytoplasmic membrane of *E. coli*^43^,^44^. EGC is reported to bind to the ATP site of the DNA gyrase b subunit of bacteria and inhibit the activity of the gyrase enzyme^43^. The bactericidal action of catechin is due to its hydrogen peroxide generation^44^. The highest antimicrobial activity of tea is due to presence of catechins polyphenols which damage the bacterial cell membrane. In terms of antimicrobial acitivity, EGC and EGCG have been shown to exhibit highest antimicrobial effect. EGCG is the most established in terms of bactericidal activity. In *Escherichia coli*, EGCG induces the destruction of biofilms by damaging bacterial membranes and degrading exopolysaccharides (EPS)^45^. Sub-MIC levels of EGCG inhibit biofilm formation in ocular Staphylococcal isolates^46^ and periodontopathogenic bacterium *Eikenella corrodens* 47. Tea catechins, including EGCG, inhibit the growth of planktonic *P. gingivalis*.^48^.

In the current study we have found that the higher the EGCG content the better the antibacterial effect against the oral microflora. GT 3 took the lead compared to the others, showing significant antibacterial activity against *S. mutans* and the human dental bacterial samples. With respect to the effect of the extract/green tea particles towards the antibacterial effect of green tea, we have presented the first time and pioneering results. From the results it is apparent that the 5K-P extracted, in the size range of 6–80 μm (from GT 1, GT 2 and GT3) do not have any distinct antibacterial property or EGCG conserved in them. Their removal from the extract in most cases led to enhanced bioactivity of the extract (5K-S). 10K-P particles were in the size range of 0.5–10 μm and these did not appear to contribute towards antimicrobial activity much. The bioactive components and EGCG contents were relatively higher compared to the 5K-P’s but their eradication did not affect the extract properties significantly. 0.5–10 μm particles may be allowed to remain in the extract. In case of the 20K-P’s which existed in the nanoregime in the GT 3 and in the smallest of the green tea particle sizes of 50 nm–6 μm, the entire story was different. They were packed with the bioactive components, namely flavanoids, total phenols, catechins, EGCG and exemplified enhanced antioxidation and antimicrobial activity compared to its counterparts. The most interesting observation made was that the antibacterial activity significantly decreased with the eradication of these particles from the extract (20K-S). These results clearly indicate that the bioactivity of green tea (represented by its antimicrobial activity in this study), was not just a green tea extract based phenomenon, but a synergistic phenomenon of the EGCG in the extract together with the EGCG packed near nano to nano sized green tea particles contained in the extract. Retention of these particles is strongly recommended. However, it needs to be reminded that the particles standalone aren’t the sole contributors, the extracts and the nanoparticles appear to equally contribute towards the bioactivity.

We also feel that these particles will also have more profound roles in the other aspects pertaining to the bioactivity of green tea (other than merely antimicrobial), which is worth future researching.

## Conclusion

In the current study, we have for the first time researched on the effect of the green tea particles suspended in the green tea extracts from commercial green tea bag infusions. The results indicated that these particles played size dependant roles in moving the antibacterial activity of the extract in the positive or negative direction. The removal of the >30 μm particles is recommended, while the retention of the nano regime particles is highly emphasized.

## Additional Information

**How to cite this article**: Gopal, J. *et al.* Bactericidal activity of green tea extracts: the importance of catechin containing nano particles. *Sci. Rep.* **6**, 19710; doi: 10.1038/srep19710 (2016).

## Figures and Tables

**Table 1:** Particle size distribution of the green tea particle obtained from FE-SEM.

Green tea particles	5K-P	10K-P	20K-P
GT 1	15–2 μm	4–10 μm	0.5–6 μm
GT 2	50–80 μm	2–10 μm	200–540 nm
GT 3	6–30 μm	0.5–3 μm	50–300 nm

## References

[b1] Chan E. W. C., Soh E. Y., Tie P. P. & Law Y. P. Antioxidant and antibacterial properties of green, black, and herbal teas of *Camellia sinensis Pharmacognosy* Research. 3, 266–272 (2011). 10.4103/0974-8490.89748 PMC3249787 22224051

[b2] Velayutham P., Babu A. & Liu D. Green tea catechins and cardiovascular health: an update. Curr. Med. Chem. 15, 1840–1850 (2008). 18691042 10.2174/092986708785132979 PMC2748751

[b3] Yue Y. *et al.* TMDB: A literature-curated database for small molecular compounds found from tea. BMC Plant Biology, 14, 243–250 (2014) 25224438 10.1186/s12870-014-0243-1 PMC4172869

[b4] Zhu Y. X., Huang H. & Tu Y. Y. A review of recent studies in China on the possible beneficial health effects of tea. Int J Food Sci Technol, 4, 333–340 (2006)

[b5] Zheng W.-J., Wan X.-C. & Bao G.-H. Brick dark tea: a review of the manufacture, chemical constituents and bioconversion of the major chemical components during fermentation. Phytochem. Rev. 14, 499–523 (2015).

[b6] Balentine S. A., Wiseman & Bouwens L. C. The chemistry of tea flavanoids. Crit. Rev. Food Sci. 37, 693–704 (1997). 10.1080/10408399709527797 9447270

[b7] Graham H. N. Green tea composition, consumption, and polyphenol chemistry. Prev. Med. 21, 334–350 (1992). 1614995 10.1016/0091-7435(92)90041-f

[b8] Chaturvedula V. S. P. & Prakash I. The aroma, taste, color and bioactive constituents of tea. J. Med. Plants Res. 5, 2110–2124 (2011), pp.

[b9] Abdel-Rahman *et al.* The safety and regulation of natural products used as foods and food ingredients. Toxicol Sci. 123, 333–348 (2011). 21821733 10.1093/toxsci/kfr198

[b10] Zaveri N. T. Green tea and its polyphenolic catechins: medicinal uses in cancer and noncancer applications. Life Sci. 78, 2073–2080 (2006). 16445946 10.1016/j.lfs.2005.12.006

[b11] Wanasundara U. N., Shahidi F. & Jablonski C. R. Comparison of standard and NMR methodologies for assessment of oxidative stability of canola and soybean oils. Food Chem. 52, 249–253 (1995).

[b12] Rice-Evans *et al.* The relative antioxidant activities of plant-derived polyphenolic flavanoids. Free Radical Res. 22, 375–383 (1995). 7633567 10.3109/10715769509145649

[b13] Johnson J. J. Bailey H. H. & Mukhtar H. Green tea polyphenols for prostate cancer chemoprevention: A translational perspective Phytomedicine. 17, 3–13. (2010) 19959000 10.1016/j.phymed.2009.09.011 PMC2789276

[b14] Juneja L. R., Chu D.-C. & Kim M. (Eds) General chemical composition of green tea and its infusion Chemistry and Applications of Green Tea. 13–22 (CRC Press Boca Raton, 1997).

[b15] Brown J. E., Khodr H., Hider R. C. & Rice-Evans C. A. Structural dependence of flavonoid interactions with Cu2^+^ ions: implications for their antioxidant properties. Biochem. J. 330, 1173–1178 (1998). 9494082 10.1042/bj3301173 PMC1219258

[b16] Hider R. C., Liu Z. D. & Khodr H. H. Metal chelation of polyphenols Method Enzymol. 335, 190–203 (2001). 10.1016/s0076-6879(01)35243-6 11400368

[b17] Kumamoto M., Sonda T., Nagayama K. & Tabata M. Effects of pH and metal ions on antioxidative activities of catechins. Biosci. Biotech. Biochem. 65, 126–132 (2001). 10.1271/bbb.65.126 11272815

[b18] Mukhtar H. & Ahmad N. Tea polyphenols: prevention of cancer and optimizing health. Am J Clin Nutr. 71, 1698S–1702S (2000). 10837321 10.1093/ajcn/71.6.1698S

[b19] Yang C. S., Maliakal P. & Meng X. Inhibition of carcinogenesis by tea. Annu Rev Pharmacol Toxicol. 42, 25–54 (2002). 11807163 10.1146/annurev.pharmtox.42.082101.154309

[b20] Cao Y. & Cao R. Angiogenesis inhibited by drinking tea. Nature, 398, 381–381 (1999). 10201368 10.1038/18793

[b21] Wang *et al.* Antimutagenic activity of green tea polyphenols. Mut. Res. 223, 273–285 (1989). 2500594 10.1016/0165-1218(89)90120-1

[b22] Han C. Screening of anticarcinogenic ingredients in tea polyphenols. Cancer Lett. 114, 153–158 (1997). 9103276 10.1016/s0304-3835(97)04647-8

[b23] Yang T. T. & Koo M. W. Inhibitory effect of Chinese green tea on endothelial cell-induced LDL oxidation. Atheroscler. 148, 67–73 (2000). 10.1016/s0021-9150(99)00239-7 10580172

[b24] Chyu, K. Y. *et al.* Differential effects of green tea-derived catechin on developing versus established atherosclerosis in apolipoprotein E-null mice. Circul. 109, 2448–2453 (2004). 10.1161/01.CIR.0000128034.70732.C2 15136500

[b25] Mandel S. & Youdim M. B. Catechin polyphenols: neurodegeneration and neuroprotection in neurodegenerative diseases. Free Radical Bio. Med. 37, 304–317 (2004). 15223064 10.1016/j.freeradbiomed.2004.04.012

[b26] Wu L. Y. *et al.* Green tea supplementation ameliorates insulin resistance and increases glucose transporter IV content in a fructose-fed rat model. Eur. J. Nut. 43, 116–124 (2004). 10.1007/s00394-004-0450-x 15083319

[b27] Stapleton P. D. *et al.* Modulation of beta-lactam resistance in Staphylococcus aureus by catechins and gallates. Int. J Antimicrob Agents. 23, 462–467 (2004). 15120724 10.1016/j.ijantimicag.2003.09.027

[b28] Nance C. L. & Shearer W. T. Is green tea good for HIV-1 infection? Aller. Clin. Immunol. 112, 851–853 (2003). 10.1016/j.jaci.2003.08.048 14610469

[b29] Esposito E. *et al.* A review of specific dietary antioxidants and the effects on biochemical mechanisms related to neurodegenerative processes. Neurobiol Aging. 23, 719–735 (2002), pp. 12392777 10.1016/s0197-4580(02)00078-7

[b30] Dona M. *et al.* Neutrophil restraint by green tea: inhibition of inflammation, associated angiogenesis, and pulmonary fibrosis. J Immunol. 170, 4335–4341 (2003). 12682270 10.4049/jimmunol.170.8.4335

[b31] Katiyar S. K. Green tea polyphenolic antioxidants and skin photoprotection. Int J Oncol. 18, 1307 (2001). 11351267 10.3892/ijo.18.6.1307

[b32] Pytkowska K. & Herbata – działanie kosmetyczne. Wiadomości PTK. **5**, 23 (2002).

[b33] Singleton V. L., Orthofer R. & Lamuela-Raventos R. M. Analysis of total phenols and other oxidation substrates and antioxidants by means of Folin-Ciocalteu reagent. Methods Enzymol. 299, 152–178 (1999).

[b34] Quettier D. C. *et al.* Phenolic compounds and antioxidant activities of buckwheat (*Fagopyrum esculentum* Moench) hulls and flour. J Ethnopharmacol. 72, 35–42 (2000). 10967451 10.1016/s0378-8741(00)00196-3

[b35] Tekao T., Watanabe N., Yagi I. & Sakata K. A simple screening method for antioxidant and isolation of several antioxidants produced by marine bacteria from fish and shellfish. Biosci. Biotechnol. Biochem. 58, 1780–1783 (1994).

[b36] Kumarasamy Y. *et al.* Screening seeds of some Scottish plants for free-radical scavenging activity. Phytother. Res. 21, 615–621 (2007) 17357975 10.1002/ptr.2129

[b37] Gopal J. *et al.* Sur. Eng. 24 (6) 447–451 (2008).

[b38] Gopal J., George R. P., Muraleedharan P. & Khatak H. S. Photocatalyst Inhibition of Microbial Adhesion by Anodized Titanium. Biofoul. 20, 167–175 (2004). 10.1080/08927010400008563 15545066

[b39] Atomssa T. & Gholap A. V. Characterization of caffeine and determination of caffeine in tea leaves using UV-visible spectrometer. *African J of Appl*. Chem. 7, 22–31 (2015).

[b40] Ramkumar P. *et al.* Formulation And Characterization Of Epigallocatechin Gallate Nanoparticles. Indo Amer. J Pharma Res. 5(01), 387–399 (2015).

[b41] Kocisko D. A. *et al.* New inhibitors of scrapie-associated prion protein formation in a library of 2000 drugs and natural products. J Virol. 77, 10288–10294 (2003). 12970413 10.1128/JVI.77.19.10288-10294.2003 PMC228499

[b42] Hubera B., Leo E., Walter F. & Jürgen P. Influence of Polyphenols on Bacterial Biofilm Formation and Quorum-sensing. Z. Naturforsch. 58c, 879–884 (2003). 10.1515/znc-2003-11-1224 14713169

[b43] Hoshino N. Kimura T., Yamaji A. & Ando T. Damage to the cytoplasmic membrane of Escherichia coli by catechin-copper (II) complexes. Free Radic Biol Med. 27, 1245–1250 (1999). 10641717 10.1016/s0891-5849(99)00157-4

[b44] Arakawa H., Maeda M., Okubo S. & Shimamura T. Role of hydrogen peroxide in bactericidal action of catechin. Biol Pharm Bull. 27, 277–281 (2004). 14993788 10.1248/bpb.27.277

[b45] Maeyama R. *et al.* Novel bactericidal surface: catechin-loaded surface-erodible polymer prevents biofilm formation. J Biomed Mater Res A. 75, 146–155 (2005). 16028232 10.1002/jbm.a.30346

[b46] Blanco A. R. *et al.*, Epigallocatechin gallate inhibitsbiofilm formation by ocular staphylococcal isolates. Antimicrob Agents Chemother 49, 4339–4343 (2005). 16189116 10.1128/AAC.49.10.4339-4343.2005 PMC1251539

[b47] Matsunaga T. *et al.* The inhibitory effects of catechins on biofilm formation by the periodontopathogenic bacterium, Eikenella corrodens. Biosci Biotechnol Biochem 74, 2445–2450 (2010). 21150103 10.1271/bbb.100499

[b48] Sakanaka S., Aizawa M., Kim M. & Yamamoto T. Inhibitory effects of green tea polyphenols on growth and cellular adherence of an oral bacterium, Porphyromonasgingivalis. Biosci Biotechnol Biochem 60, 745–749 (1996). 8704303 10.1271/bbb.60.745

